# Severe lower limb lymphoedema successfully treated with a two-stage debulking procedure: a case report

**DOI:** 10.1080/23320885.2020.1736943

**Published:** 2020-03-10

**Authors:** Adam Hague, Thomas Bragg, Melanie Thomas, Cheryl Pike, Karen Morgan, Amar Ghattaura

**Affiliations:** a The Welsh Centre for Burns and Plastic Surgery, Morriston Hospital, Swansea, UK; b Lymphoedema Network Wales, Cimla Health and Social Care Centre, Neath, UK

**Keywords:** Lymphoedema, debulking, lower limb

## Abstract

Lymphoedema is a chronic condition that has significant functional and psychosocial morbidity. We report a case of severe lower limb lymphoedema successfully treated with a two-stage debulking procedure, highlighting the significant improvements in function and quality of life this operation can have with the appropriate multidisciplinary support.

## Introduction

Lymphoedema is a chronic progressive disorder affecting up to 250 million people worldwide [1]. It is characterised by the impaired return of lymphatic fluid from the extremities resulting in oedema of the affected tissues. It can broadly be categorised into primary and secondary lymphoedema. Primary lymphoedema is caused by an inherent dysfunction in the lymphatic system whilst secondary lymphoedema results following an insult such as surgery, trauma or infection [2]. Filiriasis, a parasitic roundworm, is the leading cause of secondary lymphoedema worldwide, however, this is primarily limited to developing countries. In the developed world, the leading cause of lymphoedema is from cancer treatments including lymph node clearance [3,4]. The chronic deposition of protein-rich lymphatic fluid in the interstitial space stimulates an inflammatory reaction leading to fibrosis and further damage to the lymphatics with a resultant cycle of deterioration [5–7]. This ultimately results in a grossly enlarged extremity with increased susceptibility to infection, impairment of mobility/function and psychosocial morbidity [8–10]. Treatment therefore aims to reduce the swelling and its associated sequelae and thus improve quality of life. Various different treatment modalities exist ranging from compression garments, exercise, skin care and manual lymphatic drainage to surgical interventions.

Surgical debulking for lymphoedema was first reported as far back as 1912 when Sir Richard Charles described excision of the affected area down to deep fascia and skin grafting the defect, with resultant immediate improvements in function [11]. A similar procedure was also described by Homan, however, here skin flaps are raised and only the underlying lymphoedematous tissue is excised [12]. With recent advances in microsurgery, lymphovenous anastomosis (LVA) and vascularised lymph node transfer (VLNT) are increasing in popularity in addition to liposuction, with traditional debulking procedures acting as a last resort due to concerns regarding subsequent morbidity [13,14].

We hereby present a case of severe lower limb lymphoedema successfully treated with a two-stage radical debulking procedure, highlighting the positive impact that this operation can have on a patient’s quality of life with the appropriate multidisciplinary team (MDT) support.

## Case report

**Figure 1.**
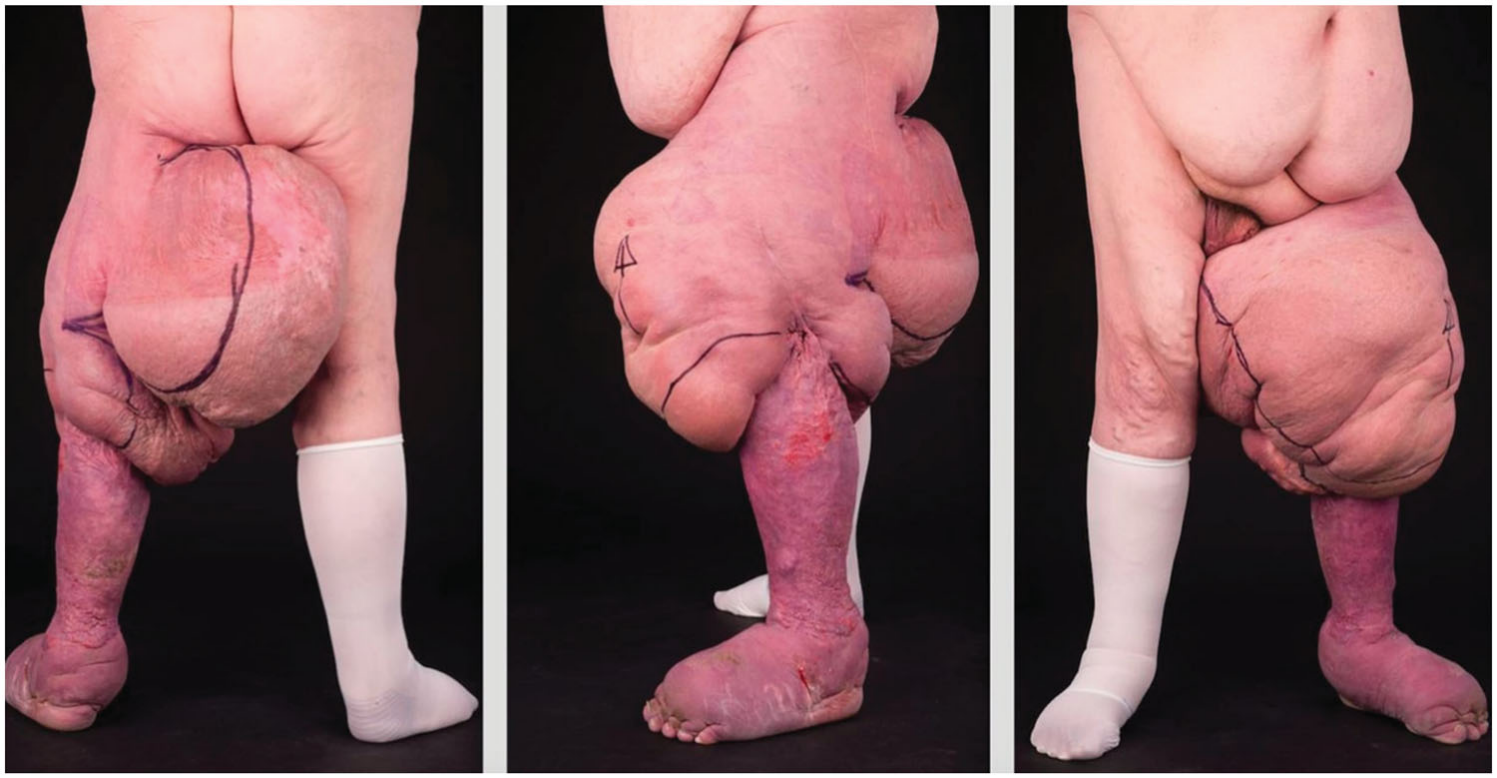
Severe lymphoedema of the left leg. Despite having hypertrophic skin medially with multiple deep folds, the skin over the lateral thigh was of good quality.

A 56-year-old gentleman was referred to our service with a long-standing history of primary lymphoedema affecting his left leg and despite being compliant, conservative management had failed to reduce the limb significantly (Figure 1). In 2010, he underwent a below-knee Charles procedure at a remote unit. Whilst this had reduced the volume of the lower leg, he was left with multiple areas of unstable skin graft and worsening swelling in his foot and thigh. The thigh swelling had become so large that he found it extremely difficult to mobilise resulting in him being house bound. He also found it difficult to pass urine due to the large mass of tissue medially, contributing to a total thigh circumference of 150 cm and the leg being 219% larger than the right with an excess volume of 25,306 millilitres (mls). His comorbidities included right-sided heart failure, sleep apnoea and atrial fibrillation. As part of his assessment an MRI scan was performed which demonstrated diffusely oedematous subcutaneous fat but no discrete cystic lesions.

After discussion at the lymphoedema MDT meeting consisting of surgeons, lymphoedema therapists and physiotherapy, the decision was made to undertake a two-stage debulking procedure of the left thigh. He was subsequently counselled carefully on the high risks that general anaesthetics presented due to his complex medical history, however, despite this, owing to the significant impact on his quality of life, he was keen to proceed with surgical intervention. An anaesthetic review was sought preoperatively.

**Figure 2.**
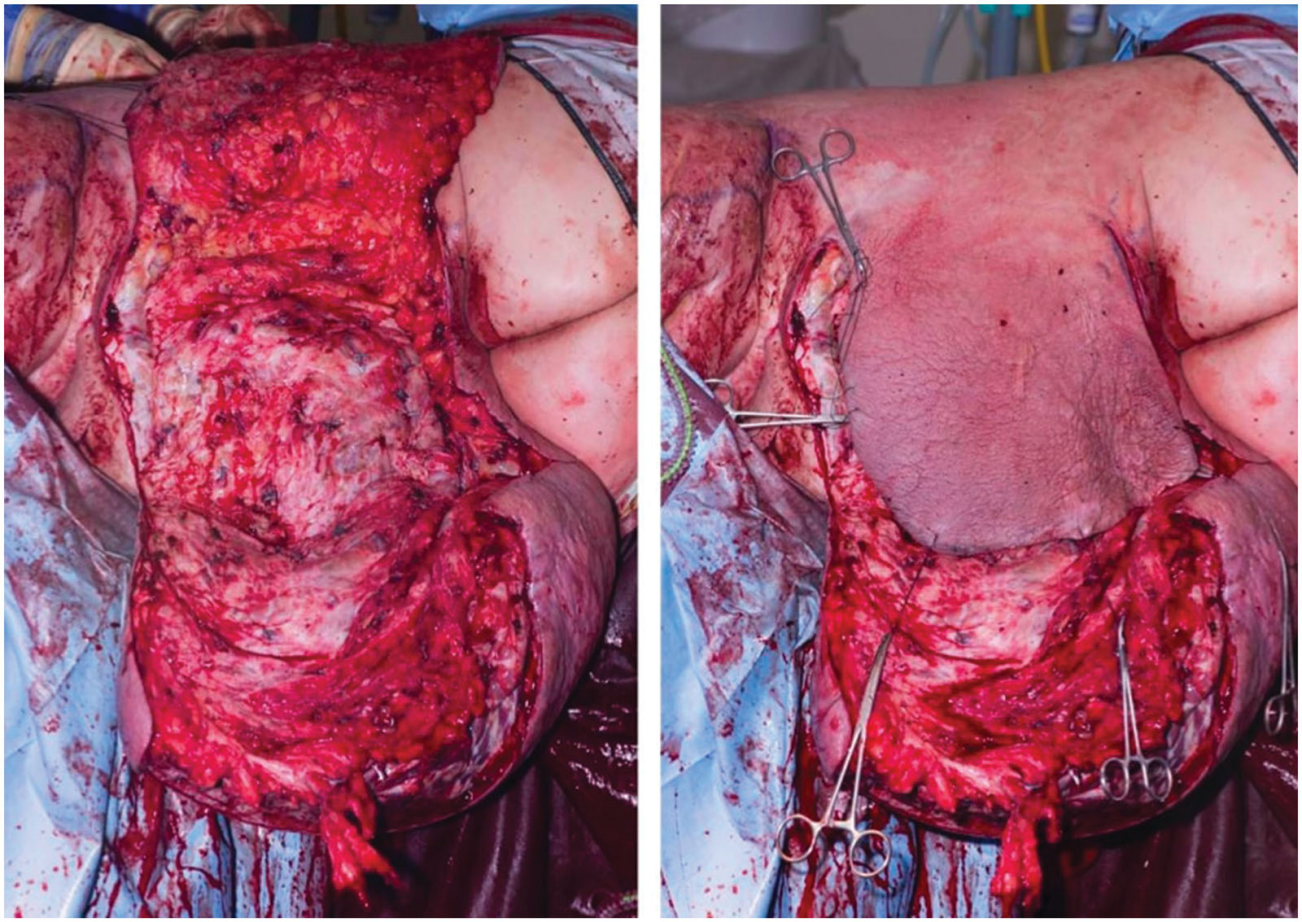
Intraoperative images of the first stage procedure demonstrating the lymphoedematous tissue to be excised and the fasciocutaneous flap to be used to cover the resultant defect.

The first procedure consisted of placing the patient in the right lateral position. Following infiltration of the area with tumescent solution, an anteriorly based fasciocutaneous flap was raised over the area of maximal thigh bulk through a medially placed incision (Figure 2). Incisions were planned in order to allow maximal debulking of the problematic area but to also facilitate closure of the wound. Lymphoedematous tissue was excised from the posterior and superolateral thigh to the knee, down to, but not including the deep fascia. Careful haemostasis was required due to the presence of numerous large traversing superficial veins. Cell salvage was also utilised with the patient receiving 2.1 litres of blood in total. Due to the large volume of blood loss intraoperatively the decision was made to not address the medial area of tissue excess during this first stage. He spent 7 days in the high dependency unit (HDU) postoperatively where he initially required vasopressor support. He was taken back to theatre 3 weeks following this initial procedure to debride and close an area of wound dehiscence around the tip of the flap. During this time the lower leg was placed in class II compression garments with wraps around the knee.

**Figure 3.**
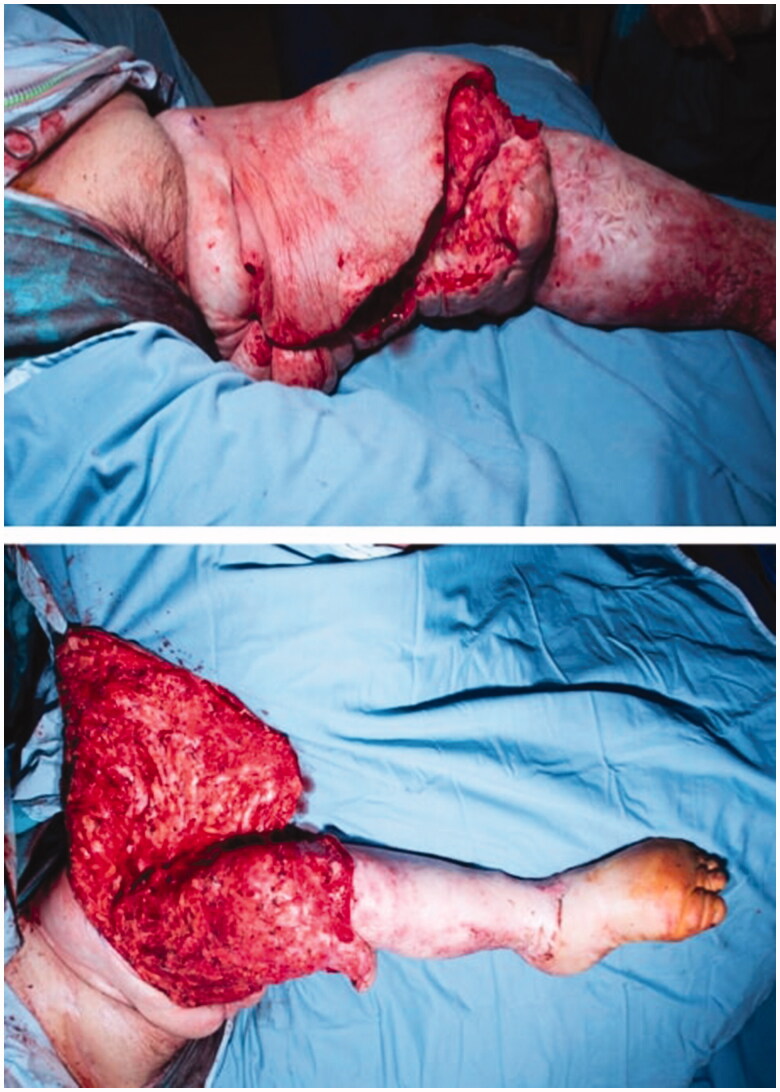
Intraoperative images during the second stage procedure. A laterally based fasciocutaneous flap was used to cover the defect.

The second stage procedure was undertaken 11 months following the first and consisted of placing the patient supine in a frog leg position. Tumescent solution was once again utilised and a laterally based flap was raised through an inferomedial incision. 3,594 grams of lymphoedematous tissue was excised primarily from the medial and anterior areas of the thigh (Figure 3). He spent 6 days in HDU and once again suffered from an area of wound dehiscence 2 weeks postoperatively. This was debrided followed by the application of a vacuum assisted closure dressing. No further operations were required. Histology from both debulking procedures demonstrated changes consistent with chronic lymphoedema.

**Figure 4.**
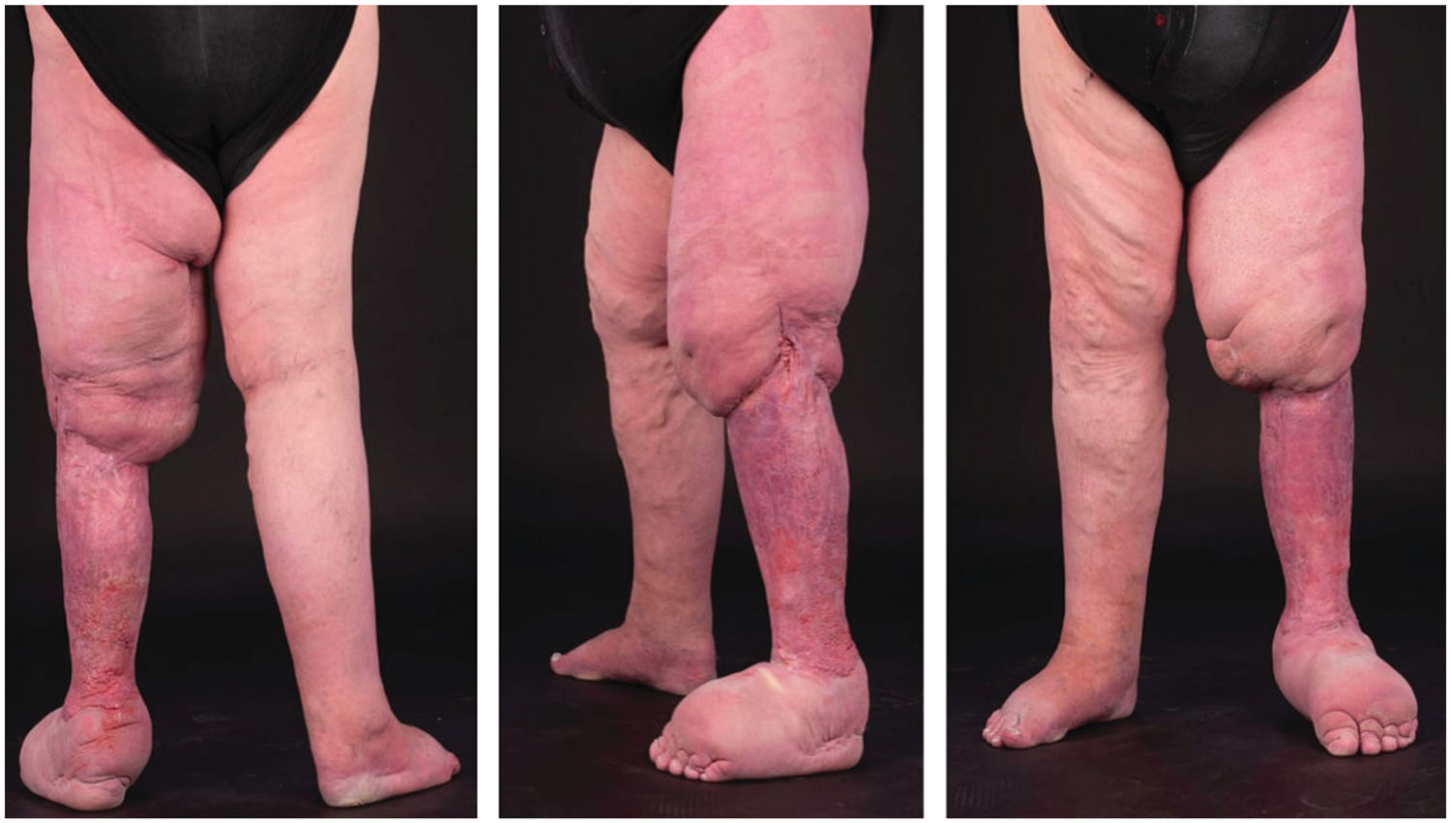
Final result 9 months following the second stage procedure demonstrating a dramatic reduction in the volume of lymphoedematous tissue.

His wounds have now completely healed and he is able to mobilise with relative ease and can pass urine without difficulty. In addition, he feels his quality of life is much improved and is more confident with the appearance of his leg (Figure 4). When compared to the contralateral side the left leg is now only 22% larger with an excess volume of 2,892 mls. To maintain this reduction he now wears 2 class II compression garments (thigh and below knee) as well as a wrapping system.

## Discussion

Lymphoedema is a debilitating condition that can have substantial physical and psychological morbidity. Treatment of these patients is extremely challenging with surgical intervention reserved for those with significant complex needs and in whom conservative measures have failed.

Various different microsurgical techniques are now available to manage lymphoedema including LVA and VLNT and these have become the surgical treatment of choice within our lymphoedema department. A smaller number of patients are offered liposuction debulking. LVA was first described in 1969 and works by diverting lymph into the venous system prior to the obstruction [15]. It is generally accepted that the earlier this procedure is performed in the disease process the more effective it is and despite numerous variations in technique being described, significant improvements have been reported [13,16–18]. VLNT is the process of transferring lymph nodes from elsewhere in the body to the lymphoedema affected area. Numerous different VLNTs have been described with promising results, including the supraclavicular flap, omental flap and groin flap [14,19,20]. The use of liposuction can remove excess adipose tissue and result in a significant reduction in volume [21].

In our patient, however, with such severe lymphoedema and thick fibrotic tissues, the only option to rapidly improve his symptoms was to use a more traditional debulking procedure. In recent times these procedures have fallen out of favour due to the associated high rates of morbidity and potential for mortality. In addition, large levels of intraoperative blood loss should be expected. Despite these risks, our case highlights the positive impact a traditional debulking procedure can have if combined with the appropriate expertise and specialist input available in a surgical lymphoedema MDT.

Due to the size of the swelling, our case was planned as a two-stage debulking to minimise the physiological insult, with a senior consultant anaesthetist and a four surgeon operating team. Utilising two consultants to undertake the excision, with trainees performing concurrent haemostasis improved operative time and minimised blood loss. The use of cell salvage as well as infiltration of the area with tumescent solution should also routinely be implemented in order to assist with this further. We recommend that all such patients should be admitted to HDU postoperatively during which time they can be provided with cardiovascular support. Postoperative wound care in both the inpatient and outpatient setting is also a vital component of these patients management. All nurses should have the appropriate training and experience in dealing with complex dressings and wounds due to lymphoedema, as postoperative delays in wound healing and infection should be expected. During their admission all patients should have physiotherapy input to address their complex mobility issues.

## Conclusion

We recommend debulking surgery in cases of severe lower limb lymphoedema where other available treatment modalities are not appropriate or have been exhausted. With the correct multidisciplinary support, significant improvements in function and quality of life can be achieved.

## References

[1] Rockson SG, Rivera KK. Estimating the population burden of lymphedema. Ann N Y Acad Sci. 2008;1131(1):147–154. 18519968 10.1196/annals.1413.014

[2] Allen RJ, Cheng MH. Lymphedema surgery: patient selection and an overview of surgical techniques. J Surg Oncol. 2016;113(8):923–931. 26846615 10.1002/jso.24170

[3] Babu S, Nutman TB. Immunopathogenesis of lymphatic filarial disease. Semin Immunopathol. 2012;34(6):847–861. 23053393 10.1007/s00281-012-0346-4 PMC3498535

[4] Cormier JN, Askew RL, Mungovan KS, et al. Lymphedema beyond breast cancer: a systematic review and meta-analysis of cancer-related secondary lymphedema. Cancer. 2010;116(22):5138–5149. 20665892 10.1002/cncr.25458

[5] Avraham T, Yan A, Zampell JC, et al. Radiation therapy causes loss of dermal lymphatic vessels and interferes with lymphatic function by TGF-beta1-mediated tissue fibrosis. Am J Physiol Cell Physiol. 2010;299:589–605. 10.1152/ajpcell.00535.2009 PMC2944320 20519446

[6] Zampell JC, Yan A, Elhadad S, et al. CD4(þ) cells regulate fibrosis and lymphangiogenesis in response to lymphatic fluid stasis. PLoS ONE. 2012;7(11):e49940. 23185491 10.1371/journal.pone.0049940 PMC3502174

[7] Cuzzone DA, Weitman ES, Albano NJ, et al. IL-6 regulates adipose deposition and homeostasis in lymphedema. Am J Physiol Heart Circ Physiol. 2014;306:1426–1434. 10.1152/ajpheart.01019.2013 PMC4024716 24633552

[8] Tobin MB, Lacey HJ, Meyer L, et al. The psychological morbidity of breast cancer-related arm swelling. Cancer. 1993;72(11):3248–3252. 8242549 10.1002/1097-0142(19931201)72:11<3248::aid-cncr2820721119>3.0.co;2-z

[9] De Godoy JMP, Braile DM, de Fatima Godoy Jr. M, et al. Quality of life and peripheral lymphedema. Lymphology. 2002;35:72–75. 12081054

[10] McWayne J, Heiney SP. Psychologic and social sequealae of secondary lymphedema: a review. Cancer. 2005;104(3):457–466. 15968692 10.1002/cncr.21195

[11] Dumanian GA, Futrell JW. The Charles procedure: misquoted and misunderstood since 1950. Plast Reconstr Surg. 1996;98(7):1258–1263. 8942914 10.1097/00006534-199612000-00022

[12] Homan J. The treatment of elephantiasis of the legs. A preliminary report. NEJM. 1936;24:1099–1104.

[13] Chang DW, Suami H, Skoracki R. A prospective analysis of 100 consecutive lymphovenous bypass cases for treatment of extremity lymphedema. Plast Reconstr Surg. 2013;132(5):1305–1314. 24165613 10.1097/PRS.0b013e3182a4d626

[14] Lin CH, Ali R, Chen SC, et al. Vascularized groin lymph node transfer using the wrist as a recipient site for management of postmastectomy upper extremity lymphedema. Plast Reconstr Surg. 2009;123(4):1265–1275. 19337095 10.1097/PRS.0b013e31819e6529

[15] Yamada Y. The studies on lymphatic venous anastomosis in lymphedema. Nagoya J Med Sci. 1969;32:1–21.

[16] O’Brien BM, Mellow CG, Khazanchi RK, et al. Long-term results after microlymphaticovenous anastomoses for the treatment of obstructive lymphedema. Plast Reconstr Surg. 1990;85:562–572. 2315396 10.1097/00006534-199004000-00011

[17] Campisi C, Bellini C, Campisi C, et al. Microsurgery for lymphedema: clinical research and long-term results. Microsurgery. 2010;30:256–260. 20235160 10.1002/micr.20737

[18] Damstra RJ, Voesten HG, van Schelven WD, et al. Lymphatic venous anastomosis (LVA) for treatment of secondary arm lymphedema. A prospective study of 11 LVA procedures in 10 patients with breast cancer related lymphedema and a critical review of the literature. Breast Cancer Res Treat. 2009;113(2):199–206. 18270813 10.1007/s10549-008-9932-5

[19] Becker C, Vasile JV, Levine JL, et al. Microlymphatic surgery for the treatment of Iatrogenic lymphedema. Clin Plast Surg. 2012;39(4):385–398. 23036289 10.1016/j.cps.2012.08.002

[20] Cheng MH, Chen SC, Henry SL, et al. Vascularized groin lymph node flap transfer for postmastectomy upper limb lymphedema: flap anatomy, recipient sites, and outcomes. Plast Reconstr Surg. 2013;131(6):1286–1298. 23714790 10.1097/PRS.0b013e31828bd3b3

[21] O'Brien BM, Khazanchi RK, Kumar PA, et al. Liposuction in the treatment of lymphoedema; a preliminary report. Br J Plast Surg. 1989;42(5):530–533. 2804517 10.1016/0007-1226(89)90039-8

